# Predicting clinical diagnosis in Huntington's disease: An imaging polymarker

**DOI:** 10.1002/ana.25171

**Published:** 2018-03-13

**Authors:** Sarah L. Mason, Richard E. Daws, Eyal Soreq, Eileanoir B. Johnson, Rachael I. Scahill, Sarah J. Tabrizi, Roger A. Barker, Adam Hampshire

**Affiliations:** ^1^ John Van Geest Centre for Brain Repair University of Cambridge United Kingdom; ^2^ Department of Clinical Neuroscience University of Cambridge United Kingdom; ^3^ The Computational, Cognitive & Clinical Neuroimaging Laboratory (CNL), Division of Brain Sciences Imperial College London United Kingdom; ^4^ Huntington's Disease Research Centre UCL Institute of Neurology, University College London United Kingdom

## Abstract

**Objective:**

Huntington's disease (HD) gene carriers can be identified before clinical diagnosis; however, statistical models for predicting when overt motor symptoms will manifest are too imprecise to be useful at the level of the individual. Perfecting this prediction is integral to the search for disease modifying therapies. This study aimed to identify an imaging marker capable of reliably predicting real‐life clinical diagnosis in HD.

**Method:**

A multivariate machine learning approach was applied to resting‐state and structural magnetic resonance imaging scans from 19 premanifest HD gene carriers (preHD, 8 of whom developed clinical disease in the 5 years postscanning) and 21 healthy controls. A classification model was developed using cross‐group comparisons between preHD and controls, and within the preHD group in relation to “estimated” and “actual” proximity to disease onset. Imaging measures were modeled individually, and combined, and permutation modeling robustly tested classification accuracy.

**Results:**

Classification performance for preHDs versus controls was greatest when all measures were combined. The resulting polymarker predicted converters with high accuracy, including those who were not expected to manifest in that time scale based on the currently adopted statistical models.

**Interpretation:**

We propose that a holistic multivariate machine learning treatment of brain abnormalities in the premanifest phase can be used to accurately identify those patients within 5 years of developing motor features of HD, with implications for prognostication and preclinical trials. Ann Neurol 2018;83:532–543

Huntington's disease (HD) is an autosomal‐dominant, fatal, neurodegenerative condition which is caused by an abnormal CAG expansion located within exon 1 of the huntingtin gene.^1^ Because of its monogenic cause, HD gene carriers can be identified before the appearance of overt clinical signs, providing a privileged window through which to observe the preclinical pathogenic pathways in HD. It also creates an opportunity to intervene before the onset of clinical disease using neuroprotective therapies or disease‐modifying drugs.

However, establishing the efficacy of any such treatment in a premanifest population presents several practical challenges. The prevalence of HD is just 12 per 100,000^2^ and with less than 1 in 5 “at‐risk” individuals undergoing predictive testing,^3^ the number of known premanifest HD gene carriers is small. Furthermore, HD is a slow progressing disease with a large variance in age of onset, especially for individuals with smaller CAG repeat lengths.^4^, ^5^ Currently, proximity to clinical diagnosis is estimated using statistical models based upon CAG repeat length and age.^4^, ^6^ However, the CAG repeat length only accounts for between 50% and 69% of the variance observed in age at diagnosis.^6^, ^7^, ^8^ Consequently, the statistical estimations of proximity to diagnosis are unreliable at the level of the individual,^5^ resulting in the need for increased sample sizes to adequately power premanifest clinical trials.

Researchers have postulated that neuroimaging markers of structural, functional, and connectivity changes in the premanifest brain have a more predictable relationship with the onset of clinically diagnosable HD.^9^, ^10^, ^11^ Over the past 5 years, the number of different imaging techniques has rapidly increased. Although each technique has its own individual merit, it is difficult to establish which one (or more than one) is the most suitable for use as a premanifest biomarker. Furthermore, the efficacy of the imaging biomarkers is judged by the strength of its relationship with the statistical estimates of proximity to diagnosis creating a circular problem.

In this study, we sought to address these issues by comparing three distinct neuroimaging measures—resting‐state functional connectivity, subcortical gray matter volume, and cortical thickness—in a population of premanifest HD gene carriers (preHD) and age‐matched controls before evaluating the utility of a novel biomarker, which combined all three. Importantly, while all participants were classified as preHD at the time of scanning, 42% received a diagnosis of clinical disease within 5 years; for these participants, real‐life time to diagnosis was also used. A multivariate machine learning approach was applied in combination with robust permutation modeling to determine the potential of each measure, for correctly classifying preHD from controls and for identifying which preHD would receive a clinical diagnosis within 5 years. Our prediction was that a holistic treatment of the data, that took into account all markers combined, would produce the most accurate clinical marker.^12^ Finally, to validate this approach we conducted an independent validation with independent structural data (functional data were not available) from the TRACK‐HD^13^ consortium.

## Patients and Methods

### Cambridge Cohort

Nineteen preHD individuals (confirmed CAG expansion) and 21 age‐matched controls were recruited from the HD clinic at the John Van Geest Centre for Brain Repair (Cambridge, UK). Ethical Approval was granted by the Local Research Ethics Committee's and informed consent was taken from participants. The preHD group was median‐split into preHD‐near and preHD‐far subgroups according to their estimated years to clinical diagnosis score calculated using the Langbehn model^4^ (median = 13.6 years). The cohort was then tracked for 5 years, during which time 8 preHD developed overt motor symptoms (HD‐converters; see Table 1 for demographics).

**Table 1:** Demographics of the Cambridge Cohort and TRACK‐HD Partial Independent Validation Cohort at Baseline

			Premanifest Baseline Classification^d^		Premanifest Follow‐up Classification^e^	
	Pre‐HD	Controls	Far	Near	Converter	Nonconverter
Cambridge cohort						
Number	19	21	9	10	8 (3^a^)	11
Age,y	45.5 (11.5)	41.9 (12.2)	41.9 (11.7)	48.7 (11.5)	50.5 (9.3)	41.9 (11.9)
Estimated years to diagnosis^c^	16.1 (8.4)	—	22.8 (7.4)	10.1 (2.7)	12.7 (7.6)	18.6 (8.4)
Disease burden score^b^	241.8 (77.2)	—	181.5 (38.1)	296.1 (60.9)	276.0 (91.8)	217.0 (56.6)
TRACK‐HD cohort						
No.	118	121	60	58	42 (20^a^)	76
Age, y	40.8 (8.9)	46.3 (10.1)	42. (11.1)	48.7 (11.5)	50.5 (9.4)	42.8 (11.8)
Estimated years to diagnosis^c^	8.1 (4.9)	—	11.5 (3.8)	4.5 (2.8)	6.0 (3.7)	7.9 (5.1)
Disease burden score^b^	274.7 (49.2)	—	237.9 (31.4)	312.8 (32.5)	295.1 (46.2)	278.6 (50.1)

a Predicted to convert within follow‐up period based upon the Langbehn^f^ equation.

b Disease burden score = age × (CAG‐35.5).

c Calculated using the Langbehn equation.^f^

d Divided by the whole Pre‐HD group median (13.6 for the Cambridge cohort; 10.8 years for the TRACK‐HD cohort).

e Division based upon the presence of a clinical diagnosis of HD at the time of follow‐up.

Taken from Langbehn DR, Brinkman RR, Falush D, Paulsen JS, Hayden MR; International Huntington's Disease Collaborative Group. A new model for prediction of the age of onset and penetrance for Huntington's disease based on CAG length. Clin Genet 2004;**65**:267–277.

### Data Acquisition

Resting‐state functional magnetic resonance imaging (fMRI; 300 T2*‐weighted volumes: repetition time = 2 seconds, echo time = 30ms, 3mm^3^ voxels) and structural (1mm^3^ magnetization‐prepared rapid gradient echo) scans were conducted using a 3 Tesla Siemens TIM Trio MRI at the MRC Cognition and Brain Sciences Unit (Cambridge, UK).

### Independent Cohort Validation

Classification models were validated with independent data from the TRACK‐HD consortium. One hundred eighteen preHD and 121 controls were recruited internationally from four sites (see earlier works^13^, ^14^ for details; Table 1). The preHD group were divided into “near” and “far” subgroups using the same methodology described above (median = 10.8 years). These independent data provided an opportunity to test the generalizability of the structural models' generalizability (fMRI data were not available).

### fMRI Processing

fMRI data were preprocessed using SPM8 (http://www.fil.ion.ucl.ac.uk/spm). Images were slice‐timing and motion corrected, coregistered to the structural image, normalized to 2mm^3^ Montreal Neurological Institute (MNI) space and spatially smoothed (8mm). Maps of canonical resting‐state networks (RSNs) were taken from a previous study^15^ and used to compare network couplings between groups. Time courses extracted^16^ for each RSN were used to calculate beta estimates for each RSN pair using two general linear models with the time course of one RSN as a dependent variable in one model and as an independent variable in the second. Individuals motion parameters and white‐matter time courses were modeled as nuisance regressors (N.B.). Motion did not differ across groups (framewise displacement [*t* = –0.0016; *p* = 0.9863); root mean square error [*t* = –0.0015; *p* = 0.3964]; spikes (*t* = 1.331; *p* = 0.1911]). Averaging across the two betas from each RSN pair produced 171 estimates of connectivity, or “coupling strengths” per individual.

### Structural Features

Estimates of cortical thickness (CT) and subcortical volumes (SCVs) were calculated using FreeSurfer (https://surfer.nmr.mgh.harvard.edu) and the Destrieux atlas.^17^

### Machine Learning

A linear support vector machine (SVM) was implemented in MATLAB (R2015b; The MathWorks, Inc., Natick, MA) and was trained to classify the preHD and control groups. For each model, the input data were standardized, age and imaging site regressed out, and normalized using a rank‐based inverse transform. Models were trained using a linear kernel, sequential minimal optimization (SMO) and a weighted cost function to account for class imbalances. Models were robustly evaluated using leave‐one‐out validation and permutation testing (1,000 iterations) of the models F1 scores, which, as the harmonic mean of the models sensitivity and precision, represents a more informative metric than classification accuracy when classes are imbalanced. Similar to classification accuracy, F1‐score chance is determined by the null distribution (∼50% in binary cases). Empirical probability values were calculated for each true model by its ranked F1 score relative to its permuted null distribution, for example, F1‐scores > 99% of the permuted models equal a *p* value < 0.01.

An independent validation was applied across the Cambridge and TRACK‐HD cohorts. Models were trained with SCVs to differentiate specific subgroups within the TRACK‐HD data set and then assessed by its F1 score when tested on the same subgroups within the Cambridge cohort against a permuted distribution (1,000 iterations).

**Figure 1:**
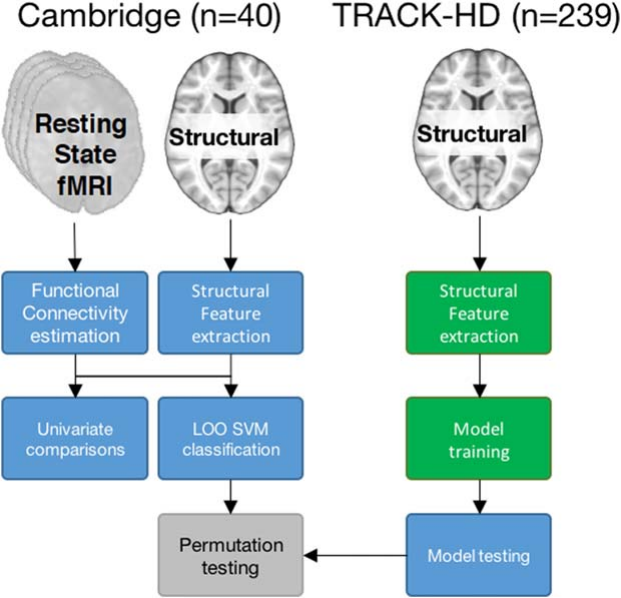
High‐level schematic of the analysis approach. In the Cambridge cohort, both resting‐state fMRI and structural images were available, only structural images were available for the TRACK‐HD cohort. Colors represent the independent samples used for different aspects of the analysis (blue = Cambridge cohort [19 preHD, 21 controls]; green = TRACK‐HD cohort [118 preHD, 121 controls]). fMRI = functional magnetic resonance imaging; SVM = support vector machine.

In summary, RSN connectivity, CT, and SCVs were compared across preHD and controls and evaluated as correlates of clinical diagnosis. These measures were then evaluated as predictors of clinical diagnosis with binary SVMs. Finally, SVMs were trained with SCVs and tested on an independent sample (see Fig 1 for a schematic of the analysis).

## Results

### Resting‐State Network Coupling

#### Cross‐Group Analysis

RSNs coupling were entered into a repeated‐measures analysis of variance (ANOVA) with Connection as the within‐participant factor (171 levels) and Group (preHD vs controls) as the between participant factor. There was a main effect of Connection (*F*_(170,6460)_= 42.264; *p* < 0.001; ηG^2^ = 0.490) and a significant Group * Connection interaction (*F*_(170,6460)_ = 1.986; *p* < 0.001; ηG^2^ = 0.043), indicating that some RSN coupling strengths differed across groups. The was no main effect of Group.

**Figure 2:**
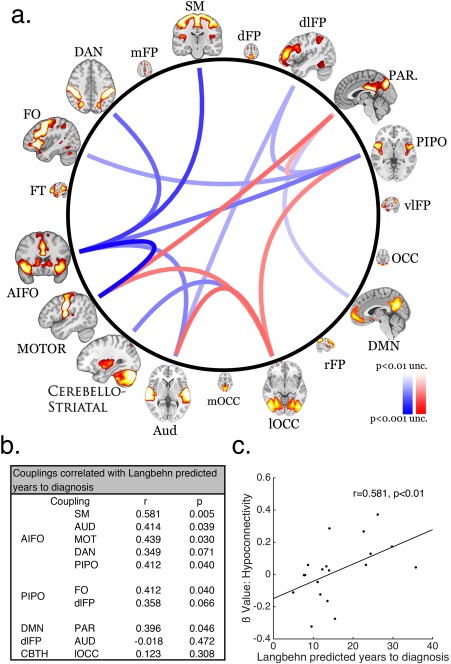
(A) Schema ball depicting cross‐group differences in resting state network coupling (connections thresholded at *p* < 0.02 uncorrected). Blue curves represent reduced network coupling and the red curves represent increased network coupling for the preHD group relative to controls. (B) Table showing correlations for the hypoconnected network measures and estimated years to diagnosis. (C) Scatterplot of the correlation between the mean values for composite hypoconnected network measures and estimated years to diagnosis.

To characterize the basis of these effects, we compared RSN couplings across groups using two‐sample *t* tests with an uncorrected two‐tailed threshold of *p* < 0.02, which identified 10 RSN connections with lower coupling strengths in preHD. Notably, a network that included the anterior insula/inferior frontal operculum (AIFO) and regions of the striatum featured in five of these reduced couplings (Fig 2A). Five couplings showed heightened network coupling at the same threshold. This approach was advantageous because it identified couplings that be averaged across to form two composite scores (hypoconnectivity and hyperconnectivity).

#### Analysis of Disease Course

Hypoconnectivity and hyperconnectivity were correlated against estimated years to clinical diagnosis (Fig 2c; N.B; this measure was not normally distributed [ks = 1; *p* < 0.001]; therefore, Spearman correlations were calculated here, and throughout). The hypoconnectivity measure showed a strong positive correlation with estimated years to clinical diagnosis^4^ (r = 0.581; p = 0.009, 95% confidence interval [CI; 0.391 0.723]) and a strong negative correlation with the CAG‐Age Product Scaled (CAPs) 18 score (r = –0.526; *p* = 0.01; 95% CI [–0.682 –0.322]). A Steiger z‐test demonstrated that estimated years to clinical diagnosis explained a significantly larger amount of variance in hypoconnectivity scores than did the CAPs measure (z = 2.568; *p* = 0.01) and was therefore not used going forward. An exploratory analysis revealed that the majority of the significant hypoconnectivity connections, including all of those with the AIFO network, showed significant positive correlations with estimated years to clinical diagnosis (Fig 2b).

Hypoconnectivity did not correlate with CAG repeat number (r = –0.241; *p* = 0.8401, 95% CI [–0.459 0.004]) or Age (r = –0.385; *p* = 0.1033; 95% CI [–0.155 –0.575]) and the relationship to estimated years to clinical diagnosis remained when age was factored out in a partial correlation (r = 0.433; *p* = 0.036, one‐tailed, 95% CI [0.210 0.612]). Finally, hyperconnectivity scores did not correlate with estimated years to clinical diagnosis (r = –0.2877; *p* = 0.2322, 95% CI [–0.497, –0.046]).

The ANOVA was repeated using the preHD subgroups to model the proximity to disease onset. There was a main effect of Connection (*F*_(170,6290)_ = 37.884; *p* < 0.001; ηG^2^ = 0.472) and a significant Group * Connection interaction (*F*_(340,6290)_ = 1.669; *p* < 0.001; ηG^2^ = 0.073). There was no main effect of Group. Because of connectivity effects going in opposing directions (as observed in the higher‐level analysis), a Tukey post‐hoc analysis revealed no group effects. However, a comparison of the hypoconnectivity composite between groups using a one‐way ANOVA revealed a main effect of Group (*F*_(2,37)_ = 15.476; *p* < 0.001). The Tukey post‐hoc analysis showed that the preHD‐near group had lower hypoconnectivity scores than the preHD‐far (*p* = 0.016; 95% CI [–0.336, –0.030]) and control (*p* < 0.001; 95% CI [–0.419, –0.163]) groups. Critically, the preHD‐far group did not differ from the control group (*p* = 0.131; 95% CI [–0.025, 0.240]). Taken together, the connectivity data demonstrate that the premanifest HD show reduced RSN coupling, primarily in networks paired with the AIFO, and these abnormalities increase as they reached clinical diagnosis.

### Subcortical Volumetrics

#### Cross‐Group Analysis

**Figure 3:**
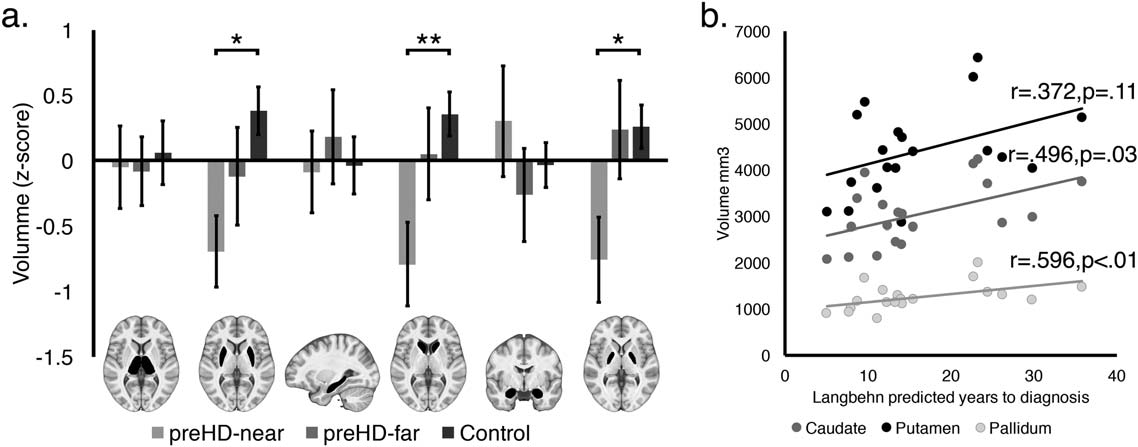
(A) Subcortical gray matter volume of the preHD‐near (light gray), preHD‐far (gray) and the matched control (light gray) groups. Each bar is accompanied by an image with the associated structure highlighted in black. Error bars report the standard error of the mean (**p < 0.01; *p < 0.05). (B) Scatterplot showing the correlation between Putamen (black dots), Caudate (gray dots), and Pallidum (light‐gray dots) volume (collapsed across hemisphere) with Estimated years to disease onset.

SCVs were entered into a repeated‐measures ANOVA with Structure (six levels) as the within‐participant factor, Group (preHD, control) as the between‐participant factor, and Age modeled as a covariate. There was a main effect of Structure (*F*_(5,185)_ = 105.645; *p* < 0.001; ηG^2^ = 0.596) and significant Structure * Group (*F*_(5,185)_ = 4.800; *p* < 0.001; ηG^2^ = 0.063) and Structure * Age (*F*_(5,185)_ = 2.740; *p* = 0.021; ηG^2^ = 0.037) interactions (Fig 3). There was no main effect of Group.

To characterize the basis of the interaction, each SCV was modeled in a one‐way ANOVA with Group (preHD‐near, preHD‐far, and controls) as the between‐participant factor. There were significant Group effects on caudate (*F*_(2,37)_ = 5.520; *p* = 0.008; η^2^ = 0.229), putamen (*F*_(2,37)_ = 4.792; *p* = 0.014; η^2^ = 0.205), and pallidum (*F*_(2,37)_ = 4.502; *p* = 0.018; η^2^ = 0.195) volumes. Post‐hoc Tukey analyses demonstrated that the preHD‐near group had significantly lower caudate (p = 0.006, 95% CI [–1,223.738, –186.021]), putamen (*p* = 0.011, 95% CI [–1,836.311, –207.492]), and pallidum (*p* = 0.018, 95% CI [–477.248, –38.329]) volumes than the controls whereas the preHD‐far and preHD‐near groups (Caudate: *p* = 0.117; 95% CI [–1,139.057 101.918]; Putamen: *p* = 0.368; 95% CI [–1,519.562, 428.295]; Pallidum: *p* = 0.061; 95% CI [–515.248, 9.641]) and the preHD‐far and control groups (Caudate: *p* = 0.678; 95% CI [–724.341, 351.720]; Putamen: *p* = 0.363, 95% CI [–1,320.771, 368.235]; Pallidum: p = 0.998; 95% CI [–232.554, 222.582]) did not differ.

#### Analysis of Disease Course

Estimated years to disease onset significantly correlated with caudate (r = 0.496; *p* = 0.031; 95% CI [0.285, 0.660]) and pallidum (r = 0.596; *p* = 0.007; 95% CI [0.411, 0.733]) volumes, whereas putamen volume did not correlate (r = 0.372; *p* = 0.117; 95% CI [0.139, 0.565]; Fig 3b). The correlation between caudate volume and estimated years to clinical diagnosis remained when age was factored out using a partial correlation (r = 0.471; *p* = 0.049; 95% CI [0.255, 0.641]). Therefore, the preHD group had reduced volumes in specific subcortical structures relative to controls, and these abnormalities became more pronounced as they approached clinical diagnosis.

### Cortical Thickness

#### Cross‐Group Analysis

CT measures were entered into a repeated‐measures ANOVA with Parcel (74 levels) and Hemisphere (Left vs Right) as the within‐participant factors, Group (preHD, control) as the between‐participant factor and Age modeled as a covariate (N.B. We observed no evidence of quadratic effects). There were main effects of Group (*F*_(1,37)_ = 5.410; *p* = 0.026; ηG^2^ = 0.024) and Parcel (*F*_(73,2701)_ = 18.970; *p* < 0.001; ηG^2^ = 0.181), and significant Group * Parcel (*F*_(73,2701)_ = 1.473; *p* = 0.006; ηG^2^ = 0.016) and Parcel * Age (*F*_(73,2701)_ = 2.022; *p* < 0.001; ηG^2^ = 0.023) interactions.

**Figure 4:**
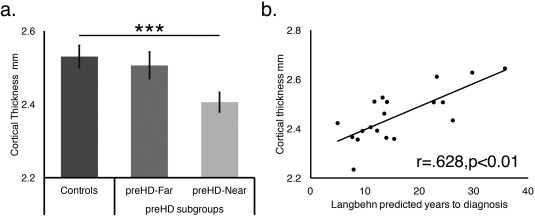
(A) Comparisons of cortical thickness (collapsed across hemisphere) between the Controls (dark gray), preHD‐far (medium gray), and preHD‐near (light gray) groups. Error bars represent the standard error of the mean (***p = 0.001). (B) A scatterplot showing the correlation between mean cortical thickness & estimated years to diagnosis.

A comparison of Mean CT across Groups using a one‐way ANOVA showed a main effect of Group (*F*_(2,37)_ = 6.132; *p* = 0.005; η^2^=0.249). A Tukey post‐hoc analysis demonstrated that mean CT was significantly lower in the preHD‐near compared to the control group (*p* = 0.004; 95% CI [–0.211, –0.036]) with a subthreshold difference compared to the preHD‐far group (*p* = 0.062; 95% CI [–0.204, 0.004]). The preHD‐far and controls did not differ (*p* = 0.797; 95% CI [–0.066 0.114]; Fig 4A).

#### Analysis of Disease Course

Estimated years to disease onset showed a positive correlation with mean CT (r = 0.628; *p* = 0.004; 95% CI [0.452, 0.756]; Fig 4B), which remained when age was factored out using a partial correlation (r = 0.579; *p* = 0.012; 95% CI [0.389, 0.721]). Therefore, the preHD group had reduced CT relative to controls and these abnormalities became more pronounced as they approached clinical diagnosis.

### Cross‐Group Classification Using a SVM

Next, determined whether the entire RSN coupling strengths, CT and SCVs feature sets could be used to classify the preHD group in a holistic manner using SVMs and permutation testing (see Materials and Methods).

We first classified the preHD and controls based on either their RSN coupling strengths, SCVs, or the CTs independently, or combined as a polymarker (Table 2, row 1). The SVMs performed significantly higher than chance for each feature set (Rest: F1 = 67%; *p* < 0.02; CT: F1 = 65%; *p* < 0.02; SCVs: F1 = 72%; *p* < 0.02; polymarker: F1 = 74%; *p* < 0.01).

**Table 2:** Classification Accuracy (%) and Comparison to Randomly Permuted Null Distribution

	LOO				External
	Rest	CT	SCV	Polymarker	SCV
Group					
preHD vs controls	**67** *	**65** *	**72** *	**74** **	**72** **
preHD‐far vs controls	49	63	48	56	48
preHD‐near vs controls	**89** ***	61	**73** *	**84** ***	**82** **
Converted HD vs controls	60	65	**88** **	**81** ***	**84** **

Table reports classification accuracy (F1 scores %) from analyses using a linear support vector machine with leave‐one‐out (LOO) and external testing. Models were assessed by their F1 scores against a randomly permuted null distribution made up of 1,000 iterations where the group labels were shuffled. The External column represents models trained on an external data set (TRACK‐HD) and tested on the Cambridge cohort. ***>=All permutations; **>=99% of permutations; *>=95% of permutations. Rest = Between Resting Network coupling. preHD = all premanifest HD individuals (N = 19). Far HD = individuals who were estimated to be far from receiving a clinical diagnosis (N = 10). Near HD = individuals who were estimated to be near to receiving a clinical diagnosis (N = 9). Converted HD = individuals who became manifest in the years between data collection and analysis (N = 8).

CT = cortical thickness; SCV = subcortical volumes.

We next observed a clear distinction in classifying the preHD‐near and far groups from controls. The preHD‐near group was successfully classified from controls (Table 2, row 3) with the Rest (F1 = 87%; *p* < 0.001), SCV (F1 = 73%; *p* < 0.05), and the polymarker (F1 = 84%; *p* < 0.001) feature sets. Conversely, the preHD‐far group was not classified from the controls with above chance accuracy (Table 2, row 2).

Finally, we classified the 8 individuals who had received clinical diagnoses in the time between data acquisition and the end of the study (Table 2, rows 4 and 5). Converted‐HD were classified from controls well above chance using the SCV (F1 = 88%; *p* < 0.01) and the polymarker (F1 = 81%; *p* < 0.001) feature sets.

### Predicting Clinical Diagnosis

#### Relationship Between HD Clinical Diagnosis and Estimated Years to Onset

**Figure 5:**
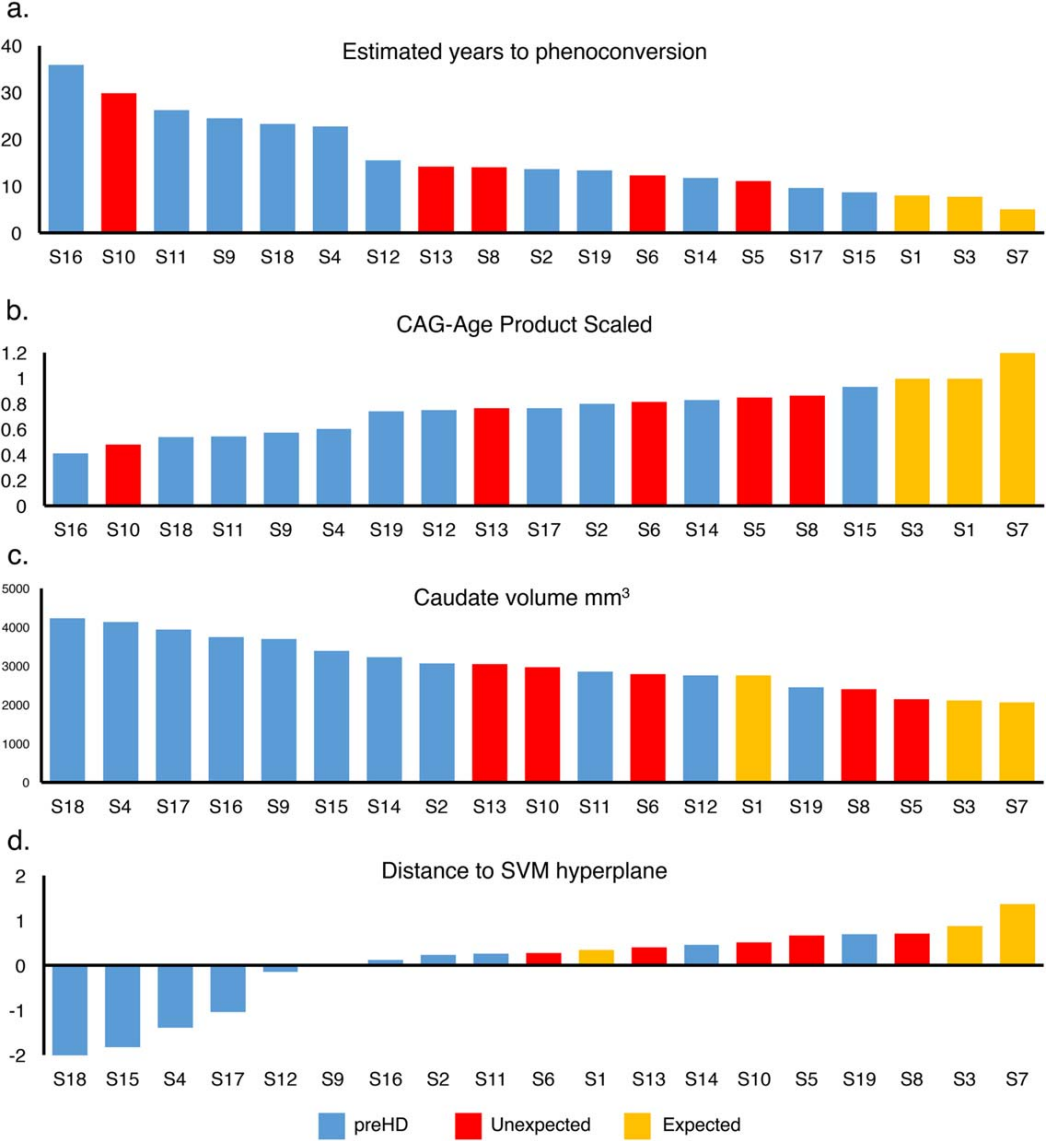
Relationship between actual time of diagnosis and estimated years to diagnosis (A), CAG‐Age product scaled (B), caudate volume (C), and SVM classification strength (D). Yellow = expected to phenoconvert within 2 years or less of the analysis date. Red = early diagnosis. Blue = yet to phenoconvert. SVM = support vector machine. Yellow = expected to phenoconvert within 5 years or less of analysis date.

Over the 5 years of this study, 5 of the Converted‐HD received clinical diagnosis despite having estimated years to onset scores that would not have predicted this (in 1 case for ∼30 years); these were labeled unexpected converters. There was no difference in the ranked estimated years to clinical diagnosis scores for the unexpected converted and the nonconverted groups (Mann–Whitney *U* = 96, *n*_1_ = 11, *n*_2_ = 5, *p* = 0.8269, HL = 1.310, 95% CI [–2.300, 8.600]; Fig 5a). Similarly, the difference in CAPs score was nonsignificant (*U* = 84, *n*_1_ = 11, *n*_2_ = 5, *p* = 0.3196, HL = –0.071, 95% CI [–0.213, –0.017]; Fig 5b).

#### Prediction of Clinical Diagnosis From Imaging Data

A pertinent question was whether the neuroimaging measures could provide an alternative prediction of disease onset. The ranked hypoconnectivity score (*U* = 90, *n*_1_ = 11, *n*_2_ = 5, *p* = 0.7427, HL = –0.113, 95% CI [–0.225, 0.027]) or mean CT (*U* = 96, *n*_1_ = 11, *n*_2_ = 5, *p* = 0.8269, HL = 0.019, 95% CI [–0.033, 0.100]) did not differ between the unexpected converted and the nonconverted groups. However, putamen (*U* = 115, *n*_1_ = 11, *n*_2_ = 5, *p* = 0.0133, HL = 1.0798e+03, 95% CI [671.700, 1.3962e+03]) and caudate volume (*U* = 113, *n*_1_ = 11, *n*_2_ = 5, *p* = 0.027, HL = 723.900, 95% CI [368.500, 988.300]) did significantly differ (Fig 5c).

Rerunning the SVM pipeline using the polymarker but directly comparing the unexpected converted and the nonconverted groups classified them with above chance accuracy (correct = 74%; accuracy, *p* < 0.03). Furthermore, distance to the classification hyperplane (a measure of classification strength) for the SVM model comparing all preHD versus controls using the polymarker feature set significantly differed between the unexpected converted and the nonconverted preHD individuals (Fig 5d; *U* = 83, *n*_1_ = 11, *n*_2_ = 5, *p* = 0.019, HL = –59.117, 95% CI [–161.632, –34.691]). Contrasting all converted individuals versus nonconverted for this measure provided a robust cross‐group difference (*U* = 86, *n*_1_ = 11, *n*_2_ = 8, *p* = 0.002, HL = –81.818, 95% CI [–170.334, –50.101]), despite the model having been blind to this information when trained. Critically, the 8 converted HD individuals were all within the top 10 when preHD participants were ranked by classification accuracy, whereas this was only the case for 3 of them when ranked by estimated years to onset. Therefore, a simple median split of the HD group based on the hyperplane distance differentiates individuals who are within 5 years of developing overt disease from those who are not with ∼84% to 89% accuracy.

#### Cross‐Cohort Validation

**Figure 6:**
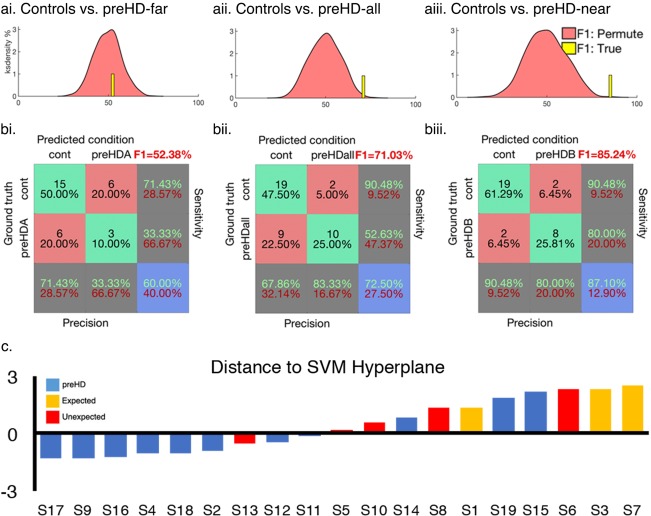
Cambridge data classified with models trained on independent data from the TRACK‐HD consortium. (A) Permuted null distribution F1 scores (pink) relative to the true model (yellow bar, N.B. Bar height and width are arbitrary) for the controls versus preHD‐far (Ai), preHD‐all subjects (Aii), and preHD‐near (Aiii) models. (B) Confusion matrices for each model. A model trained to classify preHD versus controls in the TRACK‐HD data was used to measure distance to SVM hyperplane when the model was tested on the Cambridge preHD. (C) Yellow = expected to phenoconvert within 2 years or less of the analysis date. Red = early diagnosis. Blue = yet to be diagnosed. SVM = support vector machine. Yellow = expected to phenoconvert within 5 years or less of analysis date.

In a final validation step, the SVM was trained on SCVs from an external cohort (TRACK‐HD: 118 preHD, 121 controls). The model was then tested using independent data from the Cambridge cohort (see Materials and Methods). The preHD‐far and control model performed at chance level (Fig 6A,B). However, the preHD‐all (F1 = 72%; *p* < 0.01) and preHD‐near (F1 = 82%; *p* < 0.01) versus control models performed with an above chance accuracy (Fig 6A,B). The HD‐converter model performed above chance when classifying HD‐converters within the Cambridge cohort (F1 = 84%; *p* < 0.01).

Following this, the model trained to differentiate preHD and controls using the TRACK‐HD data set was applied to the preHD subjects from the Cambridge data set. This was applied to examine how the HD‐converters and those preHD yet to convert clustered relative to the SVM hyperplane (Fig 6c). Despite the SVM being naïve to information regarding symptom onset, 87.5% of converters were assigned to the same class despite 57% of these receiving diagnoses before their expected conversion.

## Discussion

To our knowledge, this is the first study to combine brain function and structure to create a polymarker that robustly identifies whether a patient will receive a real‐life clinical diagnosis within 5 years. A consistent pattern of results emerged across imaging domains, whereby individuals predicted to be “near” to diagnosis presented robust differences relative to those predicted to be “far” from diagnosis and controls, whose performance was similar. The results also demonstrate that combining imaging metrics as a polymarker can predict whether preHD individuals are within 5 years of clinical diagnosis with greater sensitivity than the Langbehn model.^4^ Consequently, a trained classification machine of this type could be used to assign risk quotients identifying those near to diagnosis for use in clinical trial recruitment.

A strength of this work is that the SVM was trained using imaging data from participants with a definitive date of diagnosis unlike previous neuroimaging studies, where the relationship was established with a statistical estimate of proximity to diagnosis that is known to be inaccurate on an individual basis. The benefit of this is best illustrated by the relationship between classification strength and unexpected diagnosis. The Langbehn model only accurately identified 3 of the 8 individuals who did subsequently receive a clinical diagnosis, revealing a high rate of false negatives. Therefore, given the increased sensitivity, this model has the potential to be clinically useful with a greater positive predictive value than current biomarkers pending a full replication in a larger cohort.

Although never tested, it is assumed that preHD participants transition toward impairment from a normal baseline. Indeed, we have demonstrated that they have similar neural profiles to controls and consequently SVM classification was unsuccessful. This suggests that macroscopic structural and functional pathology in the preHD brain develops from a relatively normal baseline. Therefore, hypothetically treatment efficacy could be evaluated against its ability to impede the rate at which an individual's neuroimaging profile progresses toward the near‐HD classification profile, or by determining whether there is a degree of normalisation towards the profile of controls.

Although the polymarker was the most successful classifier of preHD individuals who converted from those who did not, all the imaging measures independently yielded a classification accuracy that was significantly better than chance. The most sensitive of these independent measures was subcortical volume, which classified participants with 88% accuracy. Indeed, the univariate analyses of the subcortical gray matter identified robust cross‐group differences between the preHD and controls with the volume of the caudate showing a strong relationship to the estimated time to clinical diagnosis. Crucially, when caudate volume was used to retrospectively predict those patients who were within 5 years of clinical diagnosis (Fig 5), this measure was seen to be a robust and individualized identifier of real‐life clinical diagnosis. These findings accord and extend existing work,^9^, ^19^, ^20^, ^21^ providing additional support for the use of caudate volume as a reliable estimate of disease proximity and making it a potentially useful biomarker.

Importantly, the volumetric data were also the only individual measure capable of distinguishing those participants who had received an “unexpected diagnosis” from those who remained disease free. Given the resilience of these findings, which have been demonstrated consistently across multiple studies in preHD,^22^, ^23^, ^24^, ^25^, ^26^, ^27^, ^28^, ^29^ we would suggest that analyses of subcortical volume becomes a minimum requirement for any future preclinical disease modifying trials in HD. Notably, structural volumetric analyses are likely to be more robust across scanners than resting‐state functional neuroimaging measures; therefore, they are also more likely to form a tractable basis for a standardized polymarker that can be used to integrate findings across studies and sites. This is further supported in the current study through the independent validation of the structural imaging component of the Cambridge analysis with the multisite TRACK‐HD baseline data set.^13^

Nonetheless, the functional connectivity measures contribute to the accuracy of the polymarker and provide insight into the likely basis of cognitive abnormalities in the premanifest and prodromal phases. Specifically, we observed progressive disruptions to frontostriatal system‐to‐system interactions in preHD. Altered resting‐state functional connectivity has previously been shown in preHD with reduced coupling between the left middle frontal and precentral gyrus and between the right postcentral gyrus with the medial visual network.^30^ Our findings identified a more extensive global pattern of abnormality with greater hypoconnectivity between 10 RSNs in preHD‐near than in preHD‐far and control groups. This abnormality showed a strong relationship with estimated years to clinical diagnosis.

Interestingly, interrogation of the hypoconnectivity composite revealed extensive abnormal interactions between the AIFO and other large‐scale networks in preHD. Given the progressive nature of the abnormalities observed, it is likely that degeneration within the caudate leads to abnormal modulation of AIFO function, a key node for cognitive control,^31^ and impacts on more diffuse network interactions required for executive behaviors.^32^, ^33^, ^34^ This probably relates to the executive dysfunction observed in preHD. Consequently, resting‐state fMRI may also be an appropriate biomarker for use in future therapeutic trials of potential cognitive enhancing treatments. Further work is needed to confirm the way in which resting‐state network abnormalities develop longitudinally, and how this impacts on cognitive function, especially in the light of a recent study that detected no change over a 3‐year period.^35^

Conversely, the observed hyperconnectivity between five RSNs did not robustly relate to disease course, and therefore although this they may relate to functional reorganisation in preHD, we do not consider them to be suitable biomarkers.

Finally, our analysis of the cortex detected a preHD versus control effect of reduced cortical thickness that was more pronounced in preHD individuals approaching diagnosis. Consistent with our other analyses, the preHD‐far group did not differ from controls whereas the preHD‐near group showed significantly reduced cortical thickness to both groups.

Multivariate polymarkers of the type developed here could potentially be used clinically to help preHD individuals plan their lives more securely, including employment where gene status can be a major problem (eg, military, medical profession). Moreover, disease‐modifying therapies are currently being developed with the intention of delaying the onset of clinical disease. Using this new neuroimaging polymarker should improve the selection criteria for such a study by facilitating the recruitment of participants who really have a high probability of being within 5 years of diagnosis. This is particularly relevant in an orphan disease like HD.^36^

The main limitation of this study is that our sample size was restricted by the practical limitations of recruiting from a low prevalence neurological population. However, our SVM was robustly validated using a leave‐one‐out approach relative to a permutation distribution. Additionally, we repeated the structural analysis in a larger, independently acquired data set of premanifest HD gene carriers, 36% of whom also developed clinical symptoms of the disease within 5 years, which yielded consistent results. Moreover, a major strength of our study was the longitudinal follow‐up, which allowed us to determine classification accuracy in those individuals not expected to receive a clinical diagnosis within 5 years. This was an opportunity to evaluate our model against real‐life diagnostic data and to compare its accuracy to the Langbehn model. In the cross‐validation analysis, we opted to fully replicate the methodology from the original data set including deriving a new median split within the validation cohort. This approach could be criticized because time‐to‐onset predictions should not be cohort dependent. However, we felt that it was important to respect the a priori analysis plan and, by so doing, to uphold the integrity of the analysis. In future studies, an alternative approach could be to use estimated time to disease onset as a continuous variable in a regression‐ rather than classification‐based analysis. Finally, clinical signs of HD develop across a wide range of ages; consequently, the age range for participants in this study was large (26–68 years old). Despite this, our groups and subgroups were age matched, and critical cross‐group and correlational effects were significant when age was carefully factored out.

At present, we know of only one other study that includes structural and resting MRI measures from preHD individuals (TRACK‐ON) and that could potentially be used to replicate our findings. Unfortunately, that data are currently unavailable for a replication analysis. Nonetheless, future research should replicate these results within an independent sample.

In summary, this is the first study to develop a multimodality neuroimaging polymarker of HD capable of sensitively identifying individuals who are within 5 years of their real‐life clinical diagnosis. We demonstrate the potential of multivariate statistics to outperform predictions made by the Langbehn model. Being able to identify those people who are truly “close” to diagnosis has both clinical and experimental relevance, providing both support for gene carriers who wish to work in high‐risk, high‐power professions and facilitating the most efficient and effective recruitment to future disease modifying therapeutic trials.

## Author Contributions

Concept and design of the study was undertaken by S.L.M., R.A.B., and A.H. Acquisition and analysis of the data was undertaken by S.L.M., R.E.D., E.S., E.B.J., R.I.S., S.J.T., and A.H. S.L.M., R.E.D., R.A.B., and A.H. were responsible for drafting a significant proportion of the manuscript.

## Potential Conflicts of Interest

Nothing to report.
